# Comparative evaluation of the effects of treatment with tocilizumab and TNF-α inhibitors on serum hepcidin, anemia response and disease activity in rheumatoid arthritis patients

**DOI:** 10.1186/ar4323

**Published:** 2013-10-02

**Authors:** Soken-Nakazawa J Song, Mitsuhiro Iwahashi, Naohisa Tomosugi, Kazuko Uno, Jiro Yamana, Seizou Yamana, Tomoyasu Isobe, Hiroki Ito, Hiroshi Kawabata, Kazuyuki Yoshizaki

**Affiliations:** 1 Immuno-Medical Science, Division of Applied Chemistry, Graduate School of Engineering, Osaka University, Osaka, Japan; 2 Higashihiroshima Memorial Hospital, Hiroshima, Japan; 3 Division of Nephrology, Department of Internal Medicine, Kanazawa Medical University, Ishikawa, Japan; 4 Division of Basic Research, Louis Pasteur Center for Medical Research, Kyoto, Japan; 5 Department of Hematology and Oncology, Graduate School of Medicine, Kyoto University, Kyoto, Japan

## Abstract

**Introduction:**

Anemia of inflammation (AI) is a common complication of rheumatoid arthritis (RA) and has a negative impact on RA symptoms and quality of life. Upregulation of hepcidin by inflammatory cytokines has been implicated in AI. In this study, we evaluated and compared the effects of IL-6 and TNF-α blocking therapies on anemia, disease activity, and iron-related parameters including serum hepcidin in RA patients.

**Methods:**

Patients (*n* = 93) were treated with an anti-IL-6 receptor antibody (tocilizumab) or TNF-α inhibitors for 16 weeks. Major disease activity indicators and iron-related parameters including serum hepcidin-25 were monitored before and 2, 4, 8, and 16 weeks after the initiation of treatment. Effects of tocilizumab and infliximab (anti-TNF-α antibody) on cytokine-induced hepcidin expression in hepatoma cells were analyzed by quantitative real-time PCR.

**Results:**

Anemia at base line was present in 66% of patients. Baseline serum hepcidin-25 levels were correlated positively with serum ferritin, C-reactive protein (CRP), vascular endothelial growth factor (VEGF) levels and Disease Activity Score 28 (DAS28). Significant improvements in anemia and disease activity, and reductions in serum hepcidin-25 levels were observed within 2 weeks in both groups, and these effects were more pronounced in the tocilizumab group than in the TNF-α inhibitors group. Serum hepcidin-25 reduction by the TNF-α inhibitor therapy was accompanied by a decrease in serum IL-6, suggesting that the effect of TNF-α on the induction of hepcidin-25 was indirect. In *in vitro* experiments, stimulation with the cytokine combination of IL-6+TNF-α induced weaker hepcidin expression than did with IL-6 alone, and this induction was completely suppressed by tocilizumab but not by infliximab.

**Conclusions:**

Hepcidin-mediated iron metabolism may contribute to the pathogenesis of RA-related anemia. In our cohort, tocilizumab was more effective than TNF-α inhibitors for improving anemia and normalizing iron metabolism in RA patients by inhibiting hepcidin production.

## Introduction

Rheumatoid arthritis (RA) is a chronic inflammatory autoimmune disease characterized by persistent synovitis and progressive destruction of cartilage and bones in multiple joints [1], and its most common extra-articular manifestation is anemia. Most cases of RA-associated anemia (RA-anemia) are characterized as anemia of inflammation (AI), also known as anemia of chronic disease. There is evidence, however, that RA patients with anemia have a more severe form of the disease and more serious joint damage [2-4]. Proinflammatory cytokines, particularly tumor necrosis factor α (TNF-α), interleukin 6 (IL-6) and IL-1 play important roles in the pathogenesis of RA and are thought to contribute to the development of RA-anemia by modulating iron metabolism and suppressing bone marrow erythropoiesis [5-7]. Treatments with anticytokine agents such as infliximab (anti-TNF-α), tocilizumab (anti-IL-6 receptor) and anakinra (anti-IL-1) have been shown to effectively ameliorate disease activity, inhibit joint destruction and significantly increase serum hemoglobin (Hb) levels in RA patients [8-13]. These findings suggest that the aforementioned biologic inhibitors have an antianemic effect as well as antirheumatic activities. However, the exact etiology of RA-anemia remains unclear.

Hepcidin is an antimicrobial peptide hormone synthesized mainly in the liver which has emerged as a key regulator of body iron homeostasis [14,15]. It reduces intestinal iron absorption and blocks iron release from body stores by downregulating expression of ferroportin, which is an iron exporter expressed on the surface of enterocytes and macrophages [16]. This hormone is modulated by iron homeostasis, hypoxia, erythropoiesis and inflammatory stimuli [17,18]. IL-6 is a major inducer of hepcidin expression during inflammation, and an increase in hepcidin synthesis is implicated in the etiology of AI [19,20].

We previously demonstrated that treatment with tocilizumab, by inhibiting hepcidin production, can reduce serum hepcidin and improve AI in patients with multicentric Castleman’s disease (MCD), a rare, IL-6-mediated lymphoproliferative disorder [21]. In contrast, TNF-α does not induce, but rather inhibits, hepcidin expression *in vitro*[22]. Because various cytokines are overproduced under inflammatory conditions, the control of hepcidin production in RA can be expected to be very complex. Though recent studies have found that an increase in serum hepcidin in active RA patients was associated with elevated serum IL-6 and TNF-α levels [23,24], the role of hepcidin and its regulation by cytokines in the pathogenesis of RA-anemia is still largely unknown.

To clarify these points, we evaluated and compared the effects of tocilizumab and TNF-α inhibitors on serum hepcidin levels, hematological parameters, iron status and disease activity before and after the initiation of these therapies for RA patients.

## Methods

### Patients and clinical laboratory examinations

Participants were recruited from the RA Outpatient Unit at Higashihiroshima Memorial Hospital. Patients were considered eligible if they fulfilled the American Rheumatism Association revised criteria for classification of RA [25] and their disease duration was longer than six months. Patients who had received erythropoiesis-stimulating agents (ESAs) or iron (oral or intravenous) during the two months prior to the initiation of this study were excluded. The eligible patients who had responded insufficiently to treatment with at least one disease-modifying antirheumatic drug (DMARD) or immunosuppressant were recommended for treatment with tocilizumab or TNF-α inhibitors by their attending physicians. The choice of treatment was made at the discretion of these attending physicians. From among eligible patients who started tocilizumab or TNF-α inhibitor therapy after June 2008, 50 consecutive patients in each treatment group were enrolled. Four patients in the tocilizumab group and three in the TNF-α inhibitor group dropped out of the study because of severe infection or for personal reasons. As a result, a total of 93 patients comprising 46 tocilizumab users and 47 TNF-α inhibitor users were analyzed in this study. The TNF-α inhibitor users were initiated on treatment with any of three inhibitors (etanercept, *n* = 22; infliximab, *n* = 14; or adalimumab, *n* = 11), together with oral methotrexate at a standard dose, unless the rheumatologist decided otherwise. Tocilizumab was infused in principle every four weeks at a dose of 8 mg/kg, a rate based on our previous dose determination studies [26]. Blood samples were obtained before treatment and 2, 4, 8 and 16 weeks after the initiation of treatment and then separated by centrifugation at 3,000 rpm and stored at −80°C until assayed.

Serum hepcidin-25 in all RA patients and in 16 healthy volunteers was quantified using a liquid chromatography-tandem mass spectrometry-based assay system as reported previously [21]. IL-6, TNF-α and vascular endothelial growth factor (VEGF) were measured using the Bio-Plex Suspension Array System (Bio-Rad Laboratories, Tokyo, Japan). Serum hematological parameters were measured using standard laboratory techniques. Disease Activity Score in 28 joints (DAS28) [27], used to assess patient reported outcome, was measured at baseline and at 16 weeks. This study was approved by the ethics committee of Higashihiroshima Memorial Hospital, and informed consent was obtained from all patients.

### Reagents and cell culture

Recombinant human IL-6 was provided by Ajinomoto (Kawasaki, Japan), and recombinant human TNF-α and IL-1β were purchased from Invitrogen (Carlsbad, CA, USA). Humanized anti-IL-6 receptor antibody (anti-IL6R Ab; tocilizumab) was produced and provided by Chugai Pharmaceutical Co Ltd (Tokyo, Japan), and anti-TNF-α Ab (infliximab) was provided by Mitsubishi-Tanabe Pharmaceutical Co Ltd (Tokyo, Japan). The human hepatoma cell lines PLC/PRF/5 and Hep3B were donated by Tohoku University (Sendai, Japan) and were grown in Dulbecco’s modified Eagle’s medium supplemented with 10% heat-inactivated fetal bovine serum (FBS) and 50 U/ml penicillin-streptomycin. The monocyte cell line U937 (American Type Culture Collection, Manassas, VA, USA) was cultured in RPMI 1640 medium containing 10% heat-inactivated FBS, 100 U/ml penicillin and 100 μg/ml streptomycin.

### RNA preparation and real-time RT-PCR

Cells were seeded at 5 × 10^5^ cells/well in six-well plates and stimulated with IL-6 (10 ng/ml), TNF-α (10 ng/ml) and IL-1β (10 ng/ml) under subconfluent conditions. The effect of IL-6 or TNF-α blockage was investigated by means of preincubation for 30 minutes with tocilizumab (25 μg/ml) or infliximab (25 μg/ml). To test the effect of TNF-α on IL-6 and ferroportin expression in monocytic cells, U937 cells were treated with 0.01 to 10 ng/ml recombinant TNF-α for six hours. To test the effect of TNF-α on IL-6-induced hepcidin expression in hepatocytic cells, PLC/PRF/5 and Hep3B cells were incubated with 0.01 to 10 ng/ml recombinant TNF-α for 30 minutes and then treated with IL-6. After six hours of TNF-α or IL-6 treatment, total RNA was extracted with the RNeasy Mini Kit (Qiagen GmbH, Hilden, Germany) according to the manufacturer’s instructions. cDNA was then synthesized with Moloney murine leukemia virus reverse transcriptase (Promega, Madison, WI, USA) in 25-μl reactions containing 2 μg of total RNA. Real-time quantitative RT-PCR was performed with the GeneAmp 7000 Sequence Detection System and SYBR Green dye (Applied Biosystems, Foster City, CA, USA) according to the manufacturer’s protocol. The following PCR primers were used for amplifying human hepcidin cDNA: forward primer, 5′-CCTGACCAGTGGCTCTGTTT-3′, and reverse primer, 5′CACATCCCACACTTTGATCG-3′; for amplifying human IL-6 cDNA: forward primer, 5′-ATTCGGTACATCCTCGACGGCA-3′, and reverse primer, 5′-CAGCCATCTTTGGAAGGTTCAGGT-3′; and for amplifying human ferroportin cDNA: forward primer, 5′-CCCGGAGACAAGTCCTGAATC-3′, and reverse primer, 5′-TGGCCCATTGCCACAAAGGAG-3′. The PCR was performed for a final volume of 50 μl comprising 2 × 25 μl SYBR Green PCR Master Mix (Applied Biosystems). Thermal cycle parameters were 2 minutes at 50°C and 10 minutes at 95°C, followed by 40 cycles of amplification with 15 seconds at 95°C for denaturation and 1 minute at 60°C for annealing and elongation. β_2_ microglobulin was used as an internal control for correction of hepcidin expression. Experiments were performed in triplicate, and each was repeated three times with consistent results.

### Statistical analysis

The overall effect of treatment was evaluated with the paired *t*-test on the basis of changes from baseline level observed at different intervals following tocilizumab and TNF-α inhibitor administration. Group comparisons were performed with Pearson’s correlation test. The values of serum hepcidin-25, ferritin and VEGF were log-transformed to normalize the distribution before correlation analyses. Data are expressed as means ± SD or SEM as indicated in the figure legends. *P* < 0.05 was considered significant.

## Results

### Characteristics of the study population

According to the World Health Organization definition of anemia (Hb levels lower than 13.0 g/dl for men and below 12.0 g/dl for women), 61 (66%) of the 93 active RA patients who participated in this study were anemic. Most of them showed mild anemia with a mean Hb concentration of 11.3 g/dl. The clinical characteristics of the two patient groups treated with either tocilizumab (46 cases) or TNF-α inhibitors (47 cases) are summarized in Table 1. There were no significant differences (*P* > 0.05) between the two groups for the examined variables, although the mean values for age, disease duration and CRP were slightly higher for the tocilizumab group than for the TNF-α inhibitor group. The mean level of serum hepcidin-25 in all the patients was higher (29.6 ng/ml) than that in healthy controls (20.0 ± 12.0 ng/ml), though the values varied widely. The serum Hb levels at baseline showed an inverse association with CRP (*r* = −0.45, *P* < 0.001) and DAS28 (*r* = −0.21, *P* = 0.04). The mean levels of serum ferritin and mean corpuscular volume (MCV) in all patients were normal (ferritin: 90.3 ng/ml; MCV: 93 fl), but the actual values varied widely, indicating that anemia in some cases might have originated not only from inflammation but also from other factors, including iron deficiency, folate deficiency and drug effects.

**Table 1:** **Baseline clinical characteristics of patients**^**a**^

**Characteristics**	**TCZ users (*N *** **= 46)**	**TNFi users (*N *** **= 47)**
Age (years)	60 (28 to 75)	55 (30 to 76)
Gender	3 males, 43 females	9 males, 38 females
Hb (g/dl)	11.4 (8.9 to 14.4)	11.8 (7.7 to 15.3)
MCV (fl)	91.7 (72.4 to 101.5)	94.2 (79.3 to 104.1)
Ferritin (ng/ml)	81.4 (13.5 to 285.3)	99.2 (9.8 to 398.2)
Fe (μg/dl)	45.8 (9.0 to 120.0)	56.5 (10.0 to 138.0)
CRP (mg/dl)	3.37 (0.05 to 15.24)	2.11 (0.04 to 9.16)
Hepcidin-25 (ng/ml)	28.6 (0.3 to 123)	30.5 (0.4 to 127.4)
Duration of RA (years)	12.54 (1.6 to 43)	8.16 (0.8 to 28)
SJC	7.24 (0 to 24)	5.59 (0 to 23)
TJC	6.74 (0 to 23)	6.54 (1 to 23)
RF (IU/ml)	165.54 (0 to 1,166)	144.24 (0 to 1,625)
DAS28	4.74 (2.00to 7.39)	4.45 (2.33 to 7.23)

^a^CRP, C-reactive protein; DAS28, Disease Activity Score in 28 joints; Fe, serum iron; ferritin, serum ferritin; Hb, hemoglobin; MCV, mean corpuscular volume; RF, rheumatoid factor; SJC, swollen joint count; TCZ, tocilizumab; TJC, tender joint count; TNFi, tumor necrosis factor α inhibitor. All data are means (ranges) unless otherwise specified.

### Associations of serum hepcidin with hematologic parameters, iron status and rheumatoid arthritis activity

**Figure 1:**
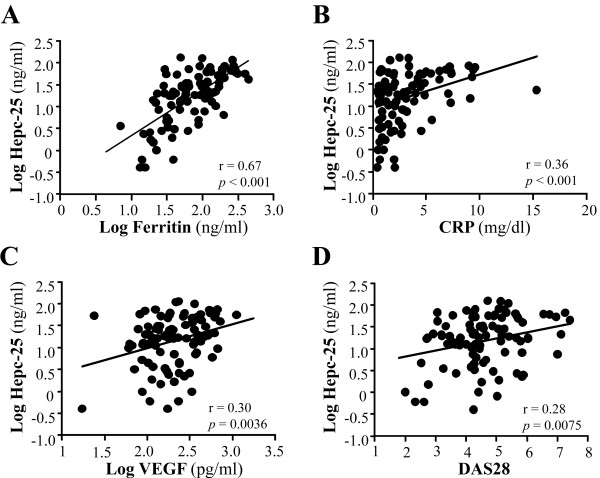
**Correlation of serum hepcidin 25 levels with serum ferritin, C-reactive protein, vascular endothelial growth factor and disease activity score in 28 joints in rheumatoid arthritis patients.** Baseline values showed a significant correlation between hepcidin-25 (Hepc-25) and **(A)** ferritin, **(B)** C-reactive protein (CRP), **(C)** vascular endothelial growth factor (VEGF) and **(D)** Disease Activity Score in 28 joints (DAS28).

**Figure 2:**
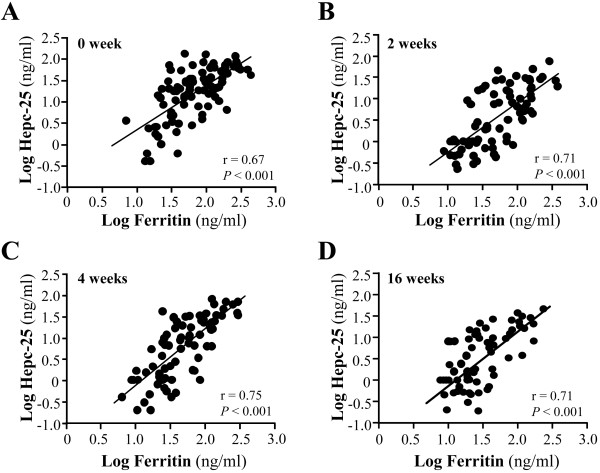
**Correlation between serum hepcidin-25 and ferritin at observation time points after biologic drug treatment.** Significant correlations were observed at **(A)** baseline, **(B)** two weeks, **(C)** four weeks and **(D)** sixteen weeks. Hepc-25, hepcidin 25.

Serum hepcidin-25 levels and the various parameters at baseline for all the patients were analyzed for possible associations. Significant and positive correlations of serum hepcidin-25 levels were found with serum ferritin (*r* = 0.67, *P* < 0.001), CRP (*r* = 0.36, *P* < 0.001) and VEGF (*r* = 0.30, *P* < 0.001) levels, as well as with the DAS28 score (*r* = 0.28, *P* < 0.001) (Figures 1A to 1D). In contrast, serum hepcidin-25 levels did not show any significant correlation with the levels of serum IL-6 (*r* = 0.15, *P* = 0.15) or TNF-α (*r* = 0.09, *P* = 0.37) or Hb (*r* = 0.006, *P* = 0.99). The most significant correlation was observed between serum hepcidin-25 and ferritin levels (*r* = 0.67) at the beginning of these treatments, and this strong and significant correlation lasted throughout the 16-week observation period (Figure 2).

### Effects of treatments with tocilizumab and tumor necrosis factor α inhibitors on serum hepcidin-25 levels

**Figure 3:**
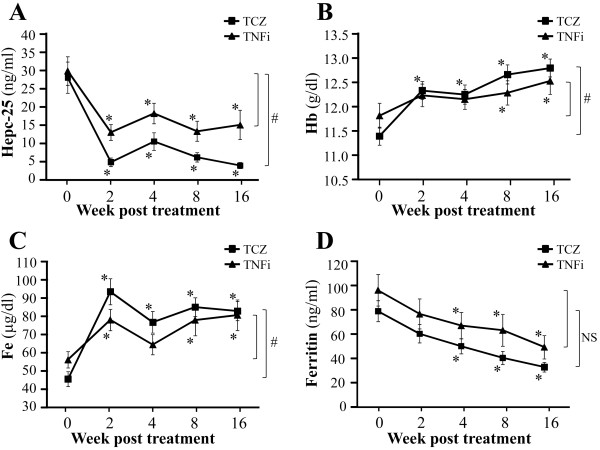
**Comparison of the effects of tocilizumab and tumor necrosis factor α inhibitors on hepcidin-25 and anemia-related factors in rheumatoid arthritis.** Treatment effects were assessed at two, four, eight and sixteen weeks after the start of biologic drug therapy. Serum hepcidin-25 was quantified using a liquid chromatography-tandem mass spectrometry-based assay system, and other parameters were measured using standard laboratory techniques. **(A)** Serum levels of hepcidin-25 (Hepc-25), **(B)** hemoglobin (Hb), **(C)** serum iron (Fe) and **(D)** serum ferritin (Ferritin). TCZ, tocilizumab; TNFi, tumor necrosis factor α inhibitors. Values are the means and SEM. ^*^P < 0.01 vs. baseline by paired *t*-test. ^#^*P* < 0.01 by paired *t*-test comparing changes from baseline between the two groups at 16 weeks. NS, not significant.

The average levels of serum hepcidin-25 before treatment between the tocilizumab and the TNF-α inhibitor groups were comparable (Table 1). Treatment with tocilizumab or TNF-α inhibitors resulted in a dramatic decrease in serum hepcidin-25 levels within two weeks, and this inhibition lasted throughout the sixteen-week observation period (*P* < 0.01 vs. baseline) (Figure 3A). The reductions in serum hepcidin-25 levels were 82%, 62%, 80% and 86% at two, four, eight and sixteen weeks, respectively, for the tocilizumab treatment group, and 56%, 39%, 55% and 50% at two, four, eight and sixteen weeks, respectively, for the TNF-α inhibitor treatment group. Tocilizumab thus displayed a significantly stronger effect than the TNF-α inhibitors on suppressing serum hepcidin-25 levels after the initiation of these treatments (*P* < 0.01) throughout the observation period (Figure 3A).

### Effects of tocilizumab and tumor necrosis factor α inhibitor treatments on hematological and iron-related parameters

The mean values of Hb, serum iron (Fe), ferritin and MCV before treatment were comparable between the tocilizumab and the TNF-α inhibitor groups. Both tocilizumab and TNF-α inhibitor therapies continually improved the anemic status of these patients. At the end of the study period, mean Hb values had increased from 11.4 to 12.8 g/dl (*P* < 0.01) in the tocilizumab group and from 11.8 to 12.5 g/dl (*P* < 0.01) in the TNF-α inhibitor group (Figure 3B). The mean Hb increase was 1.4 g/dl in the tocilizumab group, which was significantly larger than that for the TNF-α inhibitor group (0.7 g/dl) (*P* < 0.01), indicating that tocilizumab had a more beneficial effect than TNF-α inhibitors on improvement of RA-anemia. Fe gradually increased, and serum ferritin levels continually and significantly decreased, throughout the observation period in both treatment groups (Figures 3C and 3D). By the end of the study period, the mean Fe value had increased from 45.8 to 83.7 μg/dl (*P* < 0.01) in the tocilizumab group and from 56.5 to 80.4 μg/dl (*P* < 0.01) in the TNF-α inhibitor group (Figure 3C). By the end of the study period, the mean ferritin value had decreased from 81.4 to 33.9 ng/ml (*P* < 0.01) in the tocilizumab group and from 94.2 to 50.8 ng/ml (*P* < 0.01) in the TNF-α inhibitor group (Figure 3D). By the end of the study period, the mean MCV value had increased from 91.7 to 93.0 fl (*P* < 0.05) in the tocilizumab group , but the change in mean MCV value was not significant in the TNF-α inhibitor group (from 94.2 to 94.8 fl; *P* > 0.05).

### Effects of tocilizumab and tumor necrosis factor α inhibitor treatments on rheumatoid arthritis disease activity

**Figure 4:**
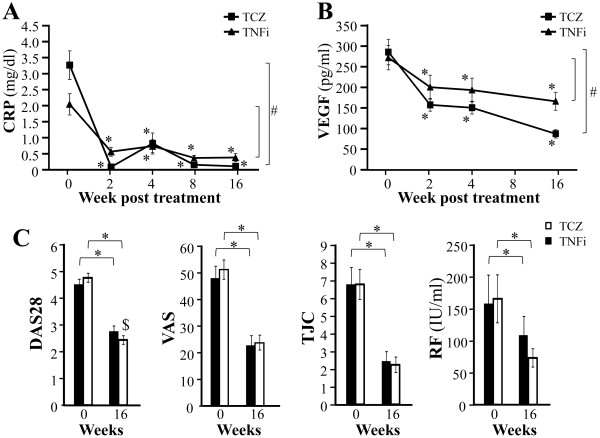
**Comparison of effects of tocilizumab and tumor necrosis factor α inhibitors on rheumatoid arthritis disease activity during 16-week treatment. (A)** C-reactive protein (CRP). **(B)** Vascular endothelial growth factor (VEGF). **(C)** Disease Activity Score in 28 joints (DAS28), tender joint count (TJC), Visual Analogue Scale (VAS) score and rheumatoid factor (RF). TCZ, tocilizumab; TNFi, tumor necrosis factor α inhibitors. Values are means and SEM. ^*^P < 0.01, vs. baseline by paired *t*-test. ^#^*P* < 0.01 and ^$^*P* < 0.05 by paired *t*-test for comparison of changes from baseline between the two groups at 16 weeks.

The initial serum levels of CRP and VEGF tended to be elevated in our patients before the anticytokine treatments. We found mean CRP levels of 3.37 and 2.21 mg/dl (reference range, <0.2 mg/dl) and mean VEGF levels of 286 and 273 pg/ml (reference range, 8.4 to 238.7 pg/ml) for the tocilizumab and TNF-α inhibitor groups, respectively. After the initiation of treatment in the tocilizumab and TNF-α inhibitor groups, mean serum CRP levels decreased to 0.08 mg/dl and 0.58 mg/dl and mean serum VEGF levels decreased to 158 pg/ml and 201 pg/ml, respectively (*P* < 0.01) (Figures 4A and 4B). All these effects continued until 16 weeks, with average values of 0.11 mg/dl for CRP and 88 pg/ml for VEGF in the tocilizumab group and average values of 0.39 mg/dl for CRP and 167 pg/ml for VEGF in the TNF-α inhibitor group (Figures 4A and 4B). At the end of the study period, both serum CRP and VEGF levels were significantly lower in the tocilizumab than in the TNF-α inhibitor group (*P* < 0.01 for CRP and VEGF).

Both tocilizumab and TNF-α inhibitor treatments also resulted in significant reductions in DAS28, Visual Analogue Scale score, tender joint count and rheumatoid factor values (Figure 4C) at 16 weeks. Although tocilizumab was more effective than TNF-α inhibitors for reducing DAS28 (*P* < 0.05), the efficacy for the other indicators was similar between these two groups.

### Interactive effects of cytokine combination on hepcidin expression *in vitro*

**Figure 5:**
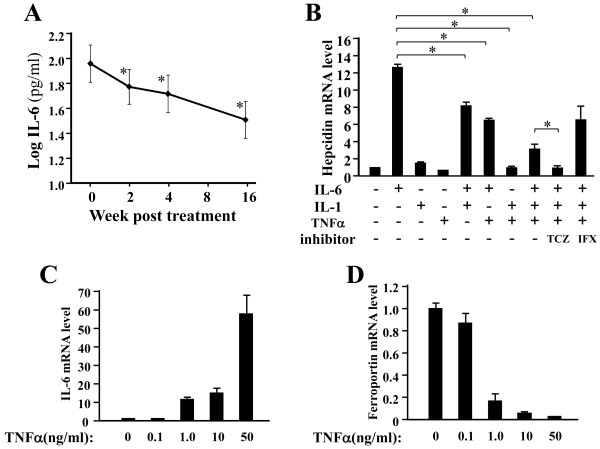
**Tumor necrosis factor α inhibitor treatment indirectly reduces hepcidin production in rheumatoid arthritis patients by reducing interleukin 6 levels. (A)** Significant reductions in mean serum interleukin 6 (IL-6) concentrations were observed at two, four and sixteen weeks after treatment with tumor necrosis factor α (TNFα) inhibitors. **(B)** Effects of tocilizumab (TCZ; 25 μg/ml) and infliximab (IFX; 25 μg/ml) on hepcidin mRNA expression induced by IL-6 (10 ng/ml), TNFα (10 ng/ml), IL-1β (10 ng/ml) and their combinations in PLC/PRF/5 cells. Hepcidin mRNA was assayed by quantitative real-time PCR six hours after the stimulation. **(C)** IL-6 mRNA expression response to TNFα stimulation was determined in U937 cells grown in medium containing TNFα at final concentrations of 0.1, 1, 10 and 50 ng/ml. IL-6 expression increased in a dose-dependent manner. **(D)** Ferroportin mRNA expression response to TNFα stimulation was determined in U937 cells grown in medium containing TNFα at final concentrations of 0.1, 1, 10 and 50 ng/ml. Ferroportin expression decreased in a dose-dependent manner. The data represent means ± SD of triplicate measurements. The same experiment was repeated at least three times with consistent results, and representative data are shown. ^*^P < 0.01 by paired *t*-test.

We found that treatment of RA patients with TNF-α inhibitors reduced serum hepcidin-25 (Figure 3A). Because IL-6 is a major inducer of hepcidin production during inflammation, we hypothesized that the reduction in serum hepcidin-25 levels in the TNF-α inhibitor users might have been the result of the decline in serum IL-6 levels. In fact, serum IL-6 levels in the TNF-α inhibitor users declined throughout the study period (Figure 5A), thus providing evidence in support of our hypothesis.

To further assess the roles of TNF-α and IL-1β in the pathogenesis of RA-anemia and their effects on IL-6-induced hepcidin mRNA expression, we performed *in vitro* real-time RT-PCR assays using a hepatoma-derived cell line. As shown in Figure 5B, the basal hepcidin mRNA levels increased by a factor of up to 12.4 at six hours after the addition of IL-6 to PLC/PRF/5 cells. TNF-α (10 ng/ml) reduced hepcidin mRNA levels by approximately 60% compared with the effect of the nonstimulated control group (Figure 5B). The hepcidin mRNA level induced by IL-6 together with TNF-α was approximately 40% lower than that induced by IL-6 alone. Similarly, the hepcidin expression level induced by IL-6 together with IL-1β was 30% lower than that induced by IL-6 alone. Hepcidin levels induced by the triple combination of IL-6, TNF-α and IL-1β were even lower than those induced by the double combination of IL-6 and TNF-α (Figure 5B). Similar results were observed in the Hep3B cell line (data not shown).

In addition, we evaluated the effect of IL-6 and TNF-α blockade on hepcidin mRNA expression by treating PLC/PRF/5 cells with tocilizumab (25 μg/ml) and infliximab (25 μg/ml) in the presence of IL-6 (10 ng/ml) together with TNF-α (10 ng/ml) and IL-1β (10 ng/ml). Tocilizumab treatment eliminated the combined effect of IL-6, TNF-α and IL-1β, so that the hepcidin expression level returned to the baseline (Figure 5B). In contrast, hepcidin expression remained elevated by the infliximab treatment (Figure 5B). We next assessed the effects of TNF-α on the mRNA expression of IL-6 and ferroportin in monocytic U937 cells. IL-6 mRNA levels were significantly upregulated, and ferroportin mRNA levels were significantly downregulated, by TNF-α in a dose-dependent manner (Figures 5C and 5D).

## Discussion

Before treatment with various biologic drugs, serum hepcidin-25 level in RA patients showed a significant positive correlation with serum ferritin, CRP and VEGF levels as well as DAS28 score. These correlations suggest that there is a link between hepcidin, abnormal iron homeostasis, inflammation and disease activity. The correlation between the serum hepcidin level and serum IL-6 level was unexpectedly not significant. This was probably due to the presence of confounding regulatory factors of hepcidin production, such as anemia and iron deficiency. We found that blocking the IL-6 or TNF-α pathway with tocilizumab or TNF-α inhibitors, respectively, significantly reduced serum hepcidin-25 levels and improved anemia and disease activity in RA patients, indicating that these cytokines are involved in both hepcidin production and the pathogenesis of RA-anemia. It is also worth noting that stronger inhibition of hepcidin-25 and significantly greater improvement in RA-anemia were observed in patients treated with tocilizumab than in patients treated with TNF-α inhibitors throughout the observation period.

Previously, we demonstrated that tocilizumab completely blocked IL-6-induced hepcidin expression in hepatoma-derived cell lines, and it effectively improved AI by inhibiting hepcidin production in MCD patients [21]. Presumably, tocilizumab plays the same role in RA-anemia. However, the mechanisms how anti-TNF-α inhibitors can improve RA-anemia is largely unknown. Unlike in MCD, multiple cytokines such as IL-6, TNF-α and IL-1 are involved in the pathogenesis of RA-anemia, and we therefore analyzed the effects of cytokine combinations on hepcidin production in hepatoma cells. As seen in Figure 5B, tocilizumab, but not infliximab, completely blocked hepcidin mRNA expression induced by the combination of IL-6+TNF-α+IL-1. We also found that TNF-α inhibited both basal level and IL-6-induced hepcidin expressions in hepatoma cells. Interestingly, however, TNF-α induced IL-6 mRNA expression in monocytic U937 cells *in vitro*. These results, taken together with the clinical finding that IL-6 levels were reduced in RA patients treated with TNF-α inhibitors, suggest that the inhibition of serum hepcidin-25 levels by TNF-α inhibitors in RA patients may be caused indirectly by a reduction in IL-6 production. These results make it clear that IL-6 is mainly responsible for hepcidin production in RA-anemia patients, which explains why tocilizumab is superior to TNF-α inhibitors for suppression of hepcidin production in RA.

However, it should be remembered that the pathogenesis of RA-anemia is complex and multifactorial. Although AI, mainly mediated by the IL-6–hepcidin axis, is considered the most frequent cause of anemia in RA, iron deficiency anemia (IDA), sometimes caused by gastrointestinal blood loss associated with the use of non-steroidal anti-inflammatory drugs, has been estimated to be the second most common form of anemia in RA. In fact, some of our patients showed relatively low serum hepcidin and ferritin levels and the anemia of such patients may be mainly attributable to iron deficiency. Therefore, hepcidin is not the only molecule playing part in bringing about this condition. It has been reported and confirmed by us that TNF-α can inhibit ferroportin mRNA expression *in vitro* and *in vivo*[28]. Ferroportin is the only cellular iron exporter in mammalian cells. In fact, TNF-α may also play an important role in RA-anemia via hepcidin-independent pathways as reported previously [12]. Another possible mechanism is that the reduction in serum hepcidin may be the result of feedback from amelioration of inflammation after treatment with TNF-α inhibitors.

Therapies using tocilizumab and TNF-α inhibitors both resulted not only in a reduction of serum hepcidin-25 levels, but also progressively normalized iron-related parameters such as serum Fe and Ferritin levels. Ferritin is a cellular storage protein for iron and elevated ferritin levels generally reflect excessive iron storage as commonly observed in AI. Serum ferritin levels in our RA patients before the treatment showed a significant correlation with serum hepcidin-25 levels, indicating an association between hepcidin and iron accumulation under RA conditions. It can thus be speculated that excessive hepcidin leads to an increase in iron storage and reduces the amount of serum iron available for Hb synthesis and erythrocyte production, which leads in turn to anemia in RA. Since the initiation of tocilizumab and TNF-α inhibitor treatments resulted in down-regulation of both serum hepcidin and ferritin levels, and since a strong correlation between serum hepcidin and ferritin was observed throughout the treatment period, it is speculated that improvement in anemia was generated by improved iron utilization through hepcidin downregulation.

Angiogenesis in synovial membranes, which is mainly regulated by VEGF, is a characteristic feature of RA patients. There is a great deal of evidence suggesting that VEGF plays an important role in the pathogenesis of RA [29]. Serum VEGF levels in RA patients correlate with disease activity scores and radiologically determined progression state [30]. Furthermore, both IL-6 and TNF-α can induce VEGF production [29,30] and anemia reportedly impacts angiogenesis via impairment of tissue oxygenation, because hypoxia is a major stimulus for the up-regulation of VEGF. Our results showed that serum hepcidin levels correlated with the serum VEGF levels in RA, suggesting that hepcidin-induced anemia can stimulate angiogenesis. We previously demonstrated that treatment with tocilizumab significantly reduces VEGF in RA [29], and in the study reported here, we confirmed that tocilizumab is more effective than TNF-α inhibitors for reducing serum VEGF.

Our finding that TNF-α inhibits both the basal level and IL-6-induced hepcidin expression in hepatocytic cell lines is consistent with that of a previous report [22]. In addition, in another preliminary study cohort comparing hepcidin-25 levels in RA and MCD patients, we found that RA patients showed significantly higher serum TNF-α and lower serum hepcidin-25 concentrations than did MCD patients (data not shown), although the two patient groups showed comparably elevated values for IL-6. Most recently, Shanmugam *et al*. [31] demonstrated that TNF-α had a negative regulatory effect on hepcidin in a mouse model of innate colitis. Therefore, our results and those of other studies suggest that TNF-α counteracts the effect of IL-6 on hepcidin production and support the notion that TNF-α may play a negative role in hepcidin production. Thus, the relatively low serum hepcidin-25 levels in patients with RA-anemia may partially be due to a balance between opposing effects of hepcidin-regulating cytokines such as IL-6 and TNF-α. However, it is also quite possible that iron deficiency and other negative regulators are involved in the downregulation of serum hepcidin levels in AI as we previously reported [21].

## Conclusions

To the best of our knowledge, this investigation is the first to compare the effects of tocilizumab and anti-TNF-α biologic drugs on RA-anemia based on changes in serum hepcidin levels. The results of our study suggest that tocilizumab is superior to TNF-α inhibitors at improving the hematologic, iron-related and inflammatory parameters in RA patients and therefore should be particularly effective for patients with severe RA-anemia.

## Abbreviations

AI: Anemia of inflammation; CRP: C-reactive protein; DAS28: Disease Activity Score in 28 joints; DMARD: Disease-modifying antirheumatic drug; ESA: Erythropoiesis-stimulating agent; Hb: Hemoglobin; IL-1: Interleukin 1; IL-6: Interleukin 6; MCD: Multicentric Castleman’s disease; MCV: Mean corpuscular volume; RA: Rheumatoid arthritis; RF: Rheumatoid factor; SJC: Swollen joint count; TCZ: Tocilizumab; TJC: Tender joint count; TNFi: Tumor necrosis factor α inhibitor; TNF-α: Tumor necrosis factor α; VAS: Visual Analogue Scale; VEGF: Vascular endothelial growth factor.

## Competing interests

The authors declare that they have no competing interests.

## Authors’ contributions

SNS designed and performed the research, analyzed the data and wrote the paper. MI, JY and SY treated and recruited the patients and analyzed the clinical data. NT and KU assisted with the data acquisition and analysis. TI and HI performed the research and analyzed the data. HK designed the research, analyzed the data, and wrote the paper. KY conceived of the study, designed the study and wrote the paper. All authors read and approved the final manuscript.
